# P-2012. Impact of Infectious Disease Consultation in the Diagnosis of Human Granulocytic Anaplasmosis at an Academic Health Care Network

**DOI:** 10.1093/ofid/ofaf695.2176

**Published:** 2026-01-11

**Authors:** Alexander Tarr, Rafael Garcia-Sturgill, George Bchech, Alexander Wrynn, Ryan Rothman, James D Como, Zaw Min, Nitin Bhanot

**Affiliations:** Allegheny Health Network, Pittsburgh, PA; Allegheny Health Network, Pittsburgh, PA; Allegheny Health Network, Pittsburgh, PA; Allegheny Health Network, Pittsburgh, PA; Allegheny General Hospital/Allegheny Health Network, Pittsburgh, Pennsylvania; Allegheny Health Network, Pittsburgh, PA; Allegheny General Hospital, Pittsburgh, Pennsylvania; Allegheny Health Network, Pittsburgh, PA

## Abstract

**Background:**

The incidence of human granulocytic anaplasmosis (HGA) has steadily increased in Pennsylvania recently. We observed that non-Infectious Disease (ID) physicians frequently did not consider this diagnosis, even when characteristic clinical and laboratory findings were present. We wanted to evaluate the impact of ID consultation on diagnosis of this infection.

**Methods:**

We conducted a retrospective chart review of adult hospitalized patients over a 5-year period (Jan 2020 – Dec 2024) using the Epic™ "SlicerDicer" tool to identify HGA cases by ICD-10 codes. Subsequently, patients who were positive on HGA serology and/or serum PCR were regarded as confirmed cases. We gathered information on how many of these patients had ID consultation placed, whether HGA was on the differential diagnosis by ID or non-ID physicians, and if there were a difference in time to appropriate antimicrobial therapy initiation.

**Results:**

Of 290 charts reviewed, 109 were included for analysis (confirmed cases). HGA was suspected in 32/109 (29%) by non-ID physicians; ID was not consulted on 12 of them. Of the ones where ID was consulted (97/109; 89%), the primary team had already considered this diagnosis in 20 patients. ID consultation identified an additional 77 patients with HGA (77/97, 79%). The average time to consultation was 1.47 hospital days. Patients with ID consultation had a mean time of 1.65 days to receive antibiotic therapy against HGA, compared to 2.5 days in those without ID involvement.

**Conclusion:**

Despite the dramatic recent increase in incidence of HGA in Pennsylvania, only in a third of our patients was this infection considered by non-ID physicians. This creates an opportunity to increase awareness amongst clinicians and aid in appropriate diagnosis. ID consultation helped identify potentially missed cases of HGA in a majority of our patients, accompanied by a reduction in time to targeted therapy.

**Disclosures:**

All Authors: No reported disclosures

